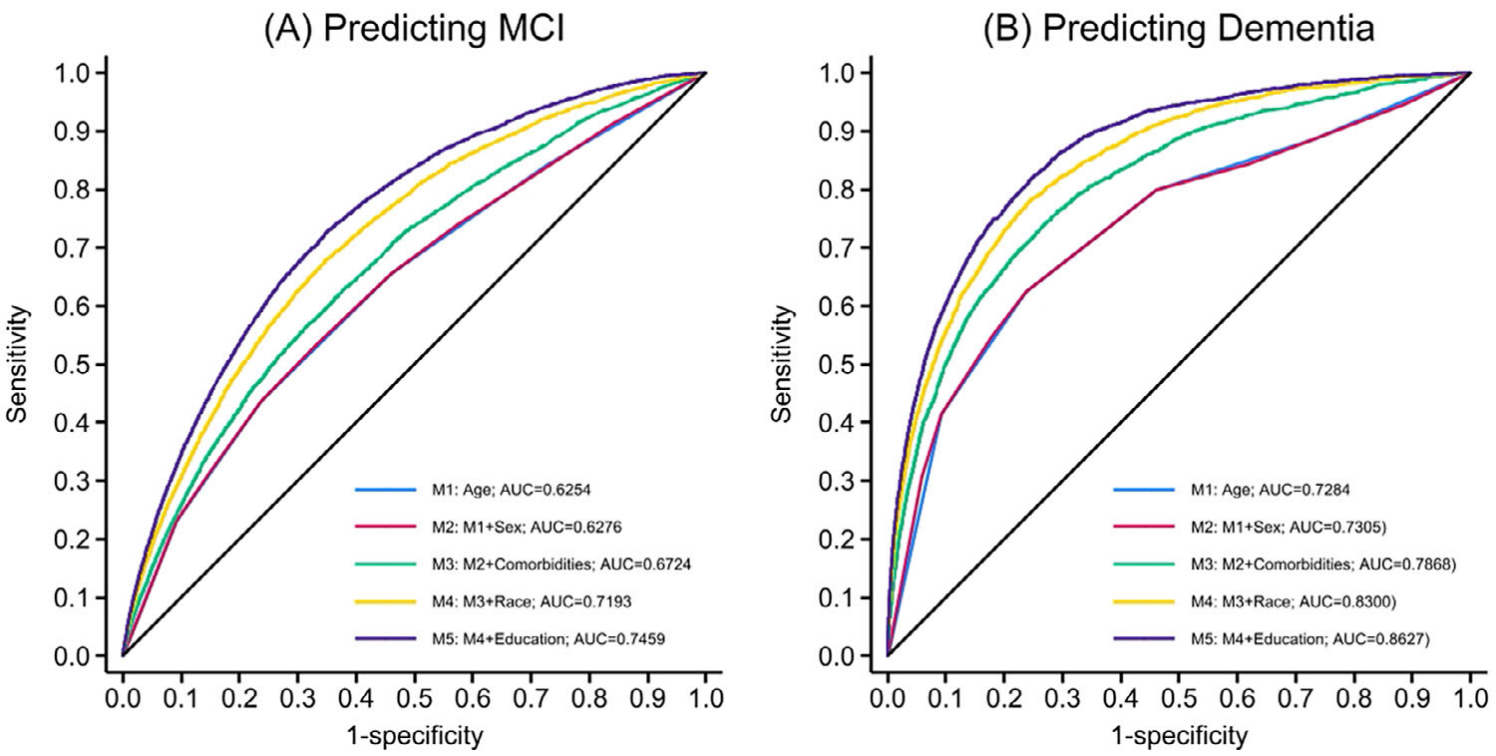# Accuracy of predicting risk of cognitive impairment using Medicare administrative data to operationalize the Lancet Commission's risk factors

**DOI:** 10.1002/alz70860_101247

**Published:** 2025-12-23

**Authors:** Wei Ye, Kate Jun, Ying Liu, Andrew Becker, Soeren Mattke

**Affiliations:** ^1^ University of Southern California, Los Angeles, CA, USA; ^2^ RAND, Boston, MA, USA

## Abstract

**Background:**

Risk factors for dementia are well established and can be used to identify individuals with higher need of cognitive evaluation, even in the absence of memory complaints. Given their ubiquitous nature, Medicare administrative data would be uniquely suited to identify such individuals. We evaluated which of the 14 risk factors identified by the 2024 Lancet Commission can be operationalized in the data and how well those predict dementia and mild cognitive impairment (MCI). The 14 risk factors are hypercholesterinemia, vision and hearing loss, hypertension, diabetes, alcohol abuse, obesity, smoking, depression, traumatic brain injury, low education, physical inactivity, social isolation, and air pollution.

**Method:**

We were able to operationalize all 10 chronic conditions based on diagnosis codes, treating COPD as proxy for smoking, and approximated education levels using geographic information. Linking Medicare data to survey responses from the Health and Retirement Study (HRS), a nationally representative, longitudinal survey of older Americans, we predicted probability of cognitive impairment based on cognitive scores in the HRS using those variables and demographic information. The model was calibrated using data from 2000 to 2014 and validated with 2016 data Model performance was assessed using the area under the receiver operating characteristic curve (AUC).

**Result:**

Age and sex predicted dementia with an AUC of 0.7305. Adding chronic conditions improved the AUC to 0.7867. Including race/ethnicity further increased the AUC to 0.8300, even after adjusting for comorbidities. Although individual‐level education contributed additional predictive value (AUC 0.8627), its absence from claims data limits its practical application. Geographic location‐based education measures, such as census‐tract‐level education, did not meaningfully contribute to prediction. Model accuracy for prediction of MCI was slightly lower (AUC: 0.7193).

**Conclusion:**

Medicare administrative data can be utilized to predict dementia and MCI risks with good accuracy. Importantly, race/ethnicity remains an independent predictor when controlling for presence of chronic conditions. As diagnosis codes have limited information on disease severity and management, this finding could point to either worse disease control or social or genetic factors. Adding person‐level information on social determinants of health to Medicare data would enhance their utility for detection of cognitive impairment.